# Vortex 6.0 all-on-chip

**DOI:** 10.1038/s41377-025-01969-w

**Published:** 2025-09-15

**Authors:** Xi Xie, Yijie Shen

**Affiliations:** 1 Centre for Disruptive Photonic Technologies, School of Physical and Mathematical Sciences, Nanyang Technological University, Singapore, Singapore; 2 School of Electrical and Electronic Engineering, Nanyang Technological University, Singapore, Singapore

**Keywords:** Micro-optics, Photonic devices, Nanophotonics and plasmonics

## Abstract

Optical vortices carrying angular momenta have promising applications from ultra-capacity communication to ultraprecise metrology, especially boosted by their recent on-chip developments. Now, the 6th-generation optical vortex technology has been unraveled by an all-on-chip integrated platform, with fully reconfigurable vector vortex control of arbitrary spin-to-orbital angular momentum coupling.

## Abstract (web summary)

Vortex 6.0: Milestone technology for all-on-chip integrated reconfigurable structured light platform.

## Graphical abstract

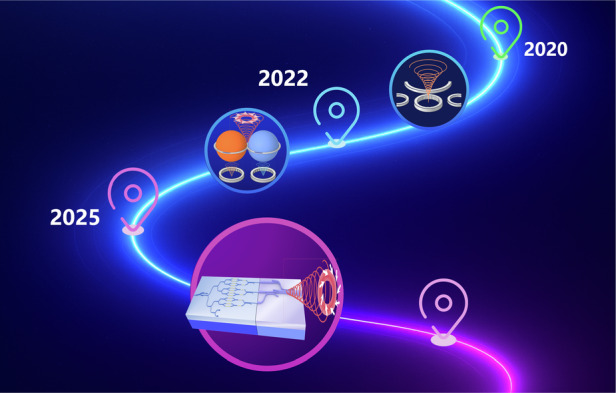

Structured light is a powerful optical paradigm that allows tailoring light’s spatial and temporal modes. This flexibility has made it a valuable tool in precision metrology, optical tweezers, and nanofabrication^1,2^. Among its various degrees of freedom (DoFs), vortex beams carrying orbital angular momentum (OAM) are unique: they possess helical wavefronts with phase singularities of arbitrary integer topological charges, in contrast to the two-level nature of polarization or spin angular momentum (SAM)^3^. This theoretically unbounded dimensionality has enabled breakthroughs in information multiplexing, quantum encoding, and precision sensing^1–3^. The realization of these applications is largely attributed to the development of miniaturized and integrated vortex beam generation devices. Over the past decades, researchers have progressed from static bulk optics to partial on-chip solutions in optical vortex beam generation, which we classify into six generations, culminating in the latest fully reconfigurable, spin-orbital coupled, all-on-chip vector vortex beam emissions.

In 1992, Allen revealed that light beams could carry OAM and proposed generating it with a pair of cylindrical lenses that endowed the beam with a helical wavefront^4^. Soon after, spiral phase plates provided a compact means of producing OAM beams^5^. However, these methods were bulky, static, imprecise and without tunability. To achieve precise mode control and flexible tunability, the computer generated digital holography was introduced which we called Vortex 2.0 technique. Building on this shift to computer-generated phase masks, liquid-crystal spatial light modulators (SLMs) soon supplanted static plates, giving experimenters a real-time knob for both the sign and magnitude of the topological charge *l*^6,7^.

The earliest two generations of approaches for vortex light sources remained static and involved the use of bulky components, which are very difficult to implement for practical applications. The challenge was revolutionized by the next two generations of on-chip vortex generators. To move beyond bulky setups, researchers turned to integrated photonics. They built compact vortex sources that emit vortex light directly from microscale even or nanoscale chips (Vortex 3.0). The first breakthrough is etching angular gratings into microring resonators, coaxing whispering-gallery modes to radiate as free-space vortices with designer topological charge^8^. A few years later, active microring lasers driven to a non-Hermitian exceptional point delivered on-chip OAM lasing with single-mode^9^. Soon after, many other non-Hermitian and topological photonic methods, for instance, the spin Hall edge mode for enhancing the modal stability of the vortex microlaser systems^10^. These micrometer-scale emitters offer high modal purity and mechanical stability, yet each chip still locks in a single, factory-set topological state. For unlocking information-encoding applications, the on-chip multiplexing, tunability and efficient detection of the vortices remained unmet needs—precisely the objectives of Vortex 4.0^11^. This generation realized two key advances: dynamic control and integrated detection of OAM beams:

(i) For dynamic control, on-chip emitters can be driven or steered on demand. For example, the photonic quantum Hall effect was involved for multiplexing large topological charges^12,13^. Applying modest magnetic field on multilayer gradient-thickness optical cavity can actively manipulate the vortex–antivortex pair^14^. All-optical control is even handier: With an optical pump flipping chirality in non-Hermitian microrings, we can generate different topological charges at exceptional points^15^. The optical pump of perovskite microcavities can also drive sub-nanosecond chirality switching^16^. All-optical switches also enabled the 1-ps-level ultrafast high-efficiency control of vortex microlasers^17^.
(ii) Detection has caught up too—WTe_2_ photodiodes turn the helical phase gradient into a winding photocurrent, revealing both the sign and strength of the OAM state^18^. Also, the on-chip photodetection was recently extended to broadband and larger range of spin and OAM based on a 2D broadband thermoelectric material (PdSe_2_)^19^.

By folding ultrafast control and direct electrical read-out onto the same chip, Vortex 4.0 turns vortex photonics from lab curiosities into a practical engine for dense optical communications and on-chip data processing.

Yet the first four generations still spoke a scalar dialect: they twisted the wavefront but left the polarization uniform. The next leap wove several DoFs into a single beam. Vortex 5.0—high-dimensional encoding braided spin-orbital coupling into vector vortex beams, adding a spatially varying polarization state atop each OAM eigenmode and thereby exploding the symbol alphabet available for data carriage. A key demonstration was the spin–orbit-coupled microlaser, which emitted coherent light in a four-dimensional Hilbert space^20^. By linking two microrings through a synthetic gauge field, the chip generated arbitrary four-component superpositions of spin-orbital modes, enabling more complex encoding schemes and richer modal control^20^. Nonetheless, the device depended on external optical pumping delivered through four separate control waveguides, demanded fine gain–phase balancing between two slightly detuned microrings, and stitched the two emissions together only in the far field—an interference-based superposition rather than a single collinear beam. These shortcomings underscored the need for a more scalable, precise, programmable, and fully integrated vortex platform, setting the stage for the new generation of vortex technology.

Such Vortex 6.0 has recently been launched: Fully reconfigurable all-on-chip vector vortex emitters. In a milestone demonstration, Zhao et al. recently unveiled a silicon platform that integrates scalar and vector OAM generation into one reprogrammable chip^21^. Unlike previous systems that relied on microrings or metasurfaces with limited tunability, this sixth-generation architecture combines a silicon-on-insulator (SOI) photonic chip with a silica multimode waveguide to synthesize arbitrary scalar and vectorial OAM beams—all generated and routed entirely on-chip^21^, see the bottom last panel of Fig. 1. At the heart of the platform is a meticulously engineered modal-control scheme. The SOI chip first splits the input light into six independent TE₀ channels; three of them are converted to TM₀ via on-chip polarization splitter–rotators, yielding three TE₀/TM₀ pairs. By independently adjusting the amplitude and phase of each channel, these six guided modes are edge-coupled into a silica mode multiplexer based on two adiabatic directional couplers, which maps them with high fidelity to six orthogonal LP modes in the multimode bus waveguide. Selective superposition of these LP modes allows on-demand generation of pure SAM beams, cylindrical vector beams or scalar/vectorial OAM beams—without any external bulk optics. Thermo-optic heaters can switch beams in ~10 µs while keeping inter-modal crosstalk below –14 dB and on-chip loss under 0.8 dB across the C-band. Because the architecture is programmable rather than resonant, it side-steps momentum-mismatch and cavity-balancing headaches and can be scaled to higher-order OAM simply by adding ports or enlarging the multimode bus. In short, Vortex 6.0 avoids the typical limitations of previous designs, such as external optical pumping, momentum-mismatch constraints or static gratings—thereby offering a low-loss, scalable route toward high-dimensional optical communications, programmable beam shaping and on-chip quantum networks.

**Fig. 1:**
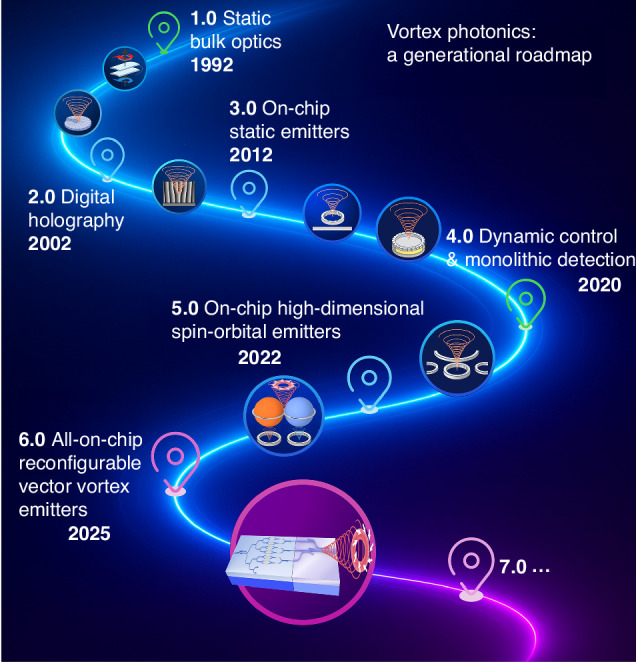
Charting the evolution of integrated vortex photonics: from early static implementations with spiral phase plates, through tunable microlasers and OAM detectors, to the latest generation of fully reconfigurable all-on-chip systems for dynamic spin-orbit coupling and high-dimensional vector vortex beam control

The road ahead points toward chips for more complex and higher-dimensional structured light that are not limited to vortex beams but steer them on demand (potential Vortex 7.0 and 8.0). For instance, a natural step is to combine electrically tunable broadband OAM lasers with on-chip nonlinear stages, so that a single system can probably lift a target beam into richer spatiotemporal vortices or spatiotemporal light fields and encode information across space-time and many dimensions of light^22^. In parallel, weaving vectorial structured light with topological mapping beyond scalar vortices could unlock topologically robust spin textures, such as skyrmions and hopfions^23,24^, offering opportunities to find their on-chip solutions. Realizing these visions will further require vortex platforms that move toward topological protection, fully integrated, system-level control of structured light. These systems must coherently shape, steer, and sense complex spin–orbit fields entirely on chip, with low loss and dynamic reconfigurability. Once this level of integration is achieved, vortex photonics will evolve from laboratory prototypes into scalable technologies for ultra-capacity communications, adaptive imaging, and robust quantum information processing.
